# AGT M235T Genotype/Anxiety Interaction and Gender in the HyperGEN Study

**DOI:** 10.1371/journal.pone.0013353

**Published:** 2010-10-13

**Authors:** Sarah S. Knox, Xinxin Guo, Yuqing Zhang, G. Weidner, Scott Williams, R. Curtis Ellison

**Affiliations:** 1 Department of Community Medicine, Mary Babb Randolf Cancer Center, West Virginia University School of Medicine, Morgontown, West Virginia, United States of America; 2 Outcome Sciences Inc., Cambridge, Massachusetts, United States of America; 3 Preventive Medicine and Epidemiology, Evans Department of Medicine, Boston University School of Medicine, Boston, Massachusetts, United States of America; 4 Department of Biology, San Francisco State University, Tiburon, California, United States of America; 5 Center for Human Genetics Research, Vanderbilt University, Nashville, Tennessee, United States of America; Universidad Europea de Madrid, Spain

## Abstract

**Background:**

Both anxiety and elevated heart rate (HR) have been implicated in the development of hypertension. The HyperGen cohort, consisting of siblings with severe and mild hypertension, an age-matched random sample of persons from the same base populations, and unmedicated adult offspring of the hypertensive siblings (N = 1,002 men and 987 women), was analyzed for an association of the angiotenisinogen AGTM235T genotype (TT, MT, MM) with an endophenotype, heart rate (HR) in high and low anxious groups.

**Methodology:**

The interaction of AGTM genotype with anxiety, which has been independently associated with hypertension, was investigated adjusting for age, hypertension status, smoking, alcohol consumption, beta blocker medication, body mass index, physical activity and hours of television viewing (sedentary life style).

**Principal Findings:**

Although there was no main effect of genotype on HR in men or women, high anxious men with the TT genotype had high HR, whereas high anxious men with the MM genotype had low HR. In women, HR was inversely associated with anxiety but there was no interaction with genotype.

**Conclusion/Significance:**

The results suggest that high anxiety in men with the TT genotype may increase risk for hypertension whereas the MM genotype may be protective in high anxious men. This type of gene x environment interaction may be one reason why genome wide association studies sometimes fail to replicate. The locus may be important only in combination with certain environmental factors.

## Introduction

Despite a great deal of research on the genetic basis of hypertension, the results have been disappointing. A genome-wide association study of 14, 000 cases and 3,000 controls reported that none of the variants previously associated with hypertension showed evidence of association [1]. One reason for this may be the presence of complex gene x environment interactions. The factors that increase or decrease disease risk of one genotype (e.g. angiotensinogen), may differ from those affecting another (e.g., the beta adrenergic receptor Arg38Gly polymorphism). If this is the case, then interactions may be one reason why relatively few genetic variants have been consistently found to contribute to complex diseases at a population level and why replication of significant findings has been difficult [2]. In addition, contributions of specific genes may be related to different points in multiple underlying causal pathways, confounding differentiation at the phenotypic level (e.g., blood pressure). These issues have led investigators to begin examining the genetic factors associated with endophenotypic precursors to the overtsymptoms of disease [2]. An endophenotype is an intermediate phenotype that may be more directly related to genotype than the clinical phenotype. An advantage of using endophenotypes is that they help to unravel the comoplexity of chronic disease by specifying intermediate gene x environment interactions that contribute to clinical endpoints.

The rennin-angiotensin system and its effects represent an important endophenotype that has been indicated by several factors. Plasma renin is involved in the physiology of blood pressure elevation and responds to environmental factors such as stress and diet. The renin-angiotensin system plays a central role in sodium and water homeostasis in the body and contributes to the maintenance of vascular tone. The AGT gene that encodes angiotensinogen is one of the prime candidates in this causal pathway. Several meta-analyses have reported that individuals with the TT allele at the site encoding amino acid residue 235 of the AGT gene (M235T genotype) have a significantly greater risk for hypertension than those with the MM genotype [3]–[6]. The 235T-allele has also been associated with CHD even after controlling for hypertension [7], indicating that it may be related to CVD risk through multiple pathways. However, the results of the studies reported so far have not been consistent [8]–[10].

If a specific genotype is associated with a disease only under certain circumstances [11], then it may not show significant association in a genome scan even if it is an important locus [12]. Interactions with other risk factors, when not taken into consideration, can lead to erroneous or contradictory conclusions. Environmental factors that influence risk for hypertension comprise a broad range of factors, e.g., diet, medications, emotionality such as anxiety and different types of stress, all of which can modulate gene expression and disease risk. Plasma renin, one component of the renin-angiotensin system and an enzyme in the pathway of the potent presser agent, Angiotensin II, has been reported to increase in response to psychological stress that also raised blood pressure and heart rate [13]. It has also been shown to increase in response to stress in mildly hypertensive men [14] on low salt diets, and to vary depending on whether the person being stressed has a family history of hypertension [15].

One of the emotional stressors that has been associated with hypertension, both in longitudinal [16], [17], [9] and cross-sectional [18] research is anxiety. However, the findings concerning association of anxiety and blood pressure are as ambiguous as those related to the M235T genotype. A possible explanation is that the association between anxiety and blood pressure is modified by genetic factors.

A review of the literature indicates that investigating individual genes or environmental exposures alone gives an incomplete and sometimes inaccurate depiction of their role in hypertension etiology [19], [20]. Because elevated blood pressure is a systemic phenomenon, an emergent property that is far downstream from the function of individual genes, researchers are beginning to investigate the association of candidate genes with endophenotypes, or precursors to hypertension to increase the power of finding an association. According to a recent review, heart rate is a significant predictor of hypertension, CHD specific and other causes of mortality [21]. Angiotensin II is also an important stress hormone [22] that is associated with increases in sympathetic activity.

The purpose of this paper was to investigate whether inconsistent results associated with the AGT M235T genotype might be related to interactions with anxiety. For reasons stated above and because most of the hypertensives in the HyperGEN cohort are medicated, we chose as our outcome measure, the endophenotype, heart rate. Due to gender differences in physiology, men and women were analyzed separately.

## Results

A total of 2195 Caucasian subjects (1,090 men, 1,105 women) had complete data on both anxiety and AGTM235T genotype. The number of MM homozygotes was 315 in men and 284 in women. There were 261 TT homozygotes in men and 249 in women, and 514 MT heterozygotes in men and 572 in women. The association between heart rate and blood pressure was significant. The average heart rate in men with hypertension was 2.7 beats/minute faster than those without hypertension (p = 0.005) and in women with hypertension average heart rate was 2.5 beats/minute faster than in normotensive women (p<0.004). Heart rate was also significantly correlated with plasma renin (r = 0.12, p = 0.0006).

The main effect of genotype on heart rate (Table 1) was not significant in either men (p = 0.86) or women (P = 0.50).

**Table 1:** Within gender mean heart rates (beats/minutes) by genotype.

Sex		AGT M235T Polymorphism			
		MM	MT	TT	P-value
Men	No. Subjects	313	488	201	
	Heart rate	65.1	64.8	65.3	P = 0.86
Women	No. Subjects	283	548	156	
	Heart rate	69.1	69.6	70.2	P = 0.50

The results for anxiety can be seen in Table 2. They show that, when examined in isolation, there is a significant association of high anxiety with lower heart rate only in women. There is no main effect of anxiety in men.

**Table 2:** Mean heart rate (beats/minute) by anxiety group in women and men.

Sex		Anxiety		
		Low	High	P-value
Men	No. Subjects	454	548	
	Heart rate	64.8	65.3	0.51
Women	No. Subjects	426	561	
	Heart rate	70.4	68.9	0.02

**Figure 1:**
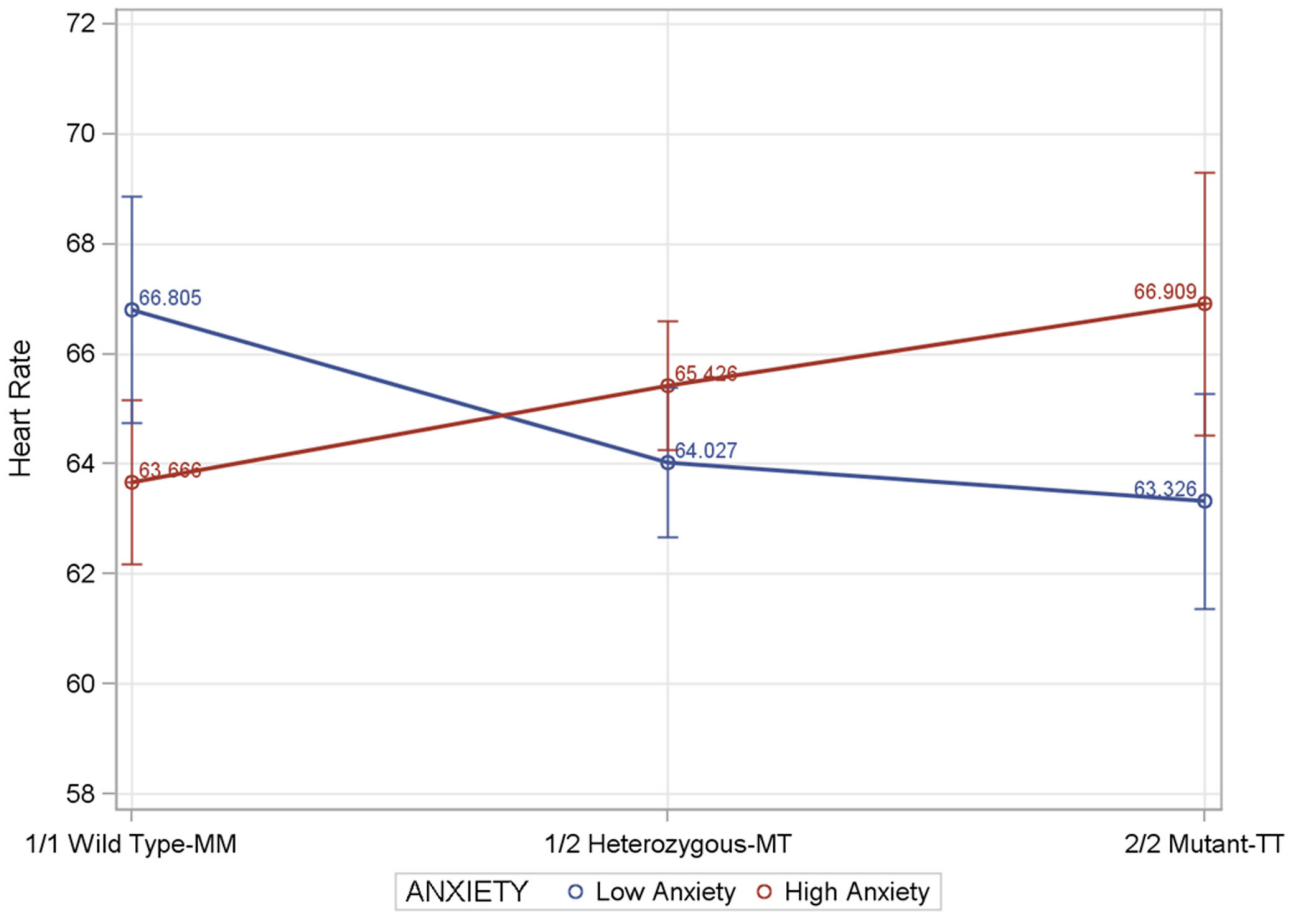
Heart Rate by Genotype and Anxiety Level in Men.

The interaction between M235T genotype and anxiety was significant in men but not in women (Table 3 and Figure 1). In men with high anxiety, the TT genotype had the highest heart rate and the MM homozygote the lowest. Average heart rate for the MT genotype fell between the average heart rates of the genotypes for the two homozygotes. These differences were significant despite the adjustment for beta blocker medication. No such pattern was present among women.

**Table 3:** Mean heart rate (beats/minute) by Anxiety group and Agtm235t genotype in women and men.

Sex	Anxiety	AGT M235T Polymorphism			
		MM	MT	TT	P-value
**Men**	Low	66.8 (n = 143)	64.0 (n = 219)	63.3 (n = 92)	0.003
	High	63.7 (n = 170)	65.4 (n = 269)	66.9 (n = 109)	
**Women**	Low	70.2 (n = 131)	70.1 (n = 244)	71.3 (n = 51)	0.78
	High	68.1 (n = 152)	69.0 (n = 304)	69.3 (n = 105)	

## Discussion

After adjusting for confounders, this population based study revealed a lack of association between AGT M235T genotype and heart rate in men and women when analyzed in the absence of data on anxiety. However, when the interaction of genotype with anxiety was examined, significant findings emerged. Among men with high anxiety, the TT genotype had the highest heart rate and the MM genotype the lowest. Given that almost all hypertensives are medicated, it is not surprising that the absolute values of heart rate are not high. However, the significant variation of heart rate by genotype in men in the presence of anxiety indicates that the TT genotype would be more at risk in the presence of anxiety than either the MM homozygote or the heterzygote. No such interaction was observed in women. The inverse association between HR and anxiety in women suggests that there are other environmental factors involved that were not investigated in this study. Our findings indicate that the effect of a single gene may be masked by gene x environment (in this case, anxiety) interactions, and may lead to false negative conclusions about genetic factors related to pathophysiologic mechanisms.

The widespread inconsistency reported in many candidate gene studies [23], [24] has variously been attributed to factors such as epistasis, population drift, population stratification, and genetic diversity. More than 50 genes associated with heart rhythm have been identified in mice [25], and differences by sex and strain of animals have also been found [26]. In the HyperGEN Study, Wilk et al [27] found that the proportion of variance in resting heart rate explained by genetic factors was 24% in African-Americans and 27% for whites. Given this evidence for epistasis in the endophenotype of heart rate and given the number of endophenotypes that may be involved in sustained blood pressure elevation, it is little wonder that genome wide association studies of hypertension have been difficult to replicate. In addition to epistasis, the field of epigenetics has also taught us that gene expression can be up and down regulated by cues from the micro-environment, which means that one and the same gene may be associated with different phenotypes depending on the environmental influences to which it is being exposed at the time of measurement. The current study seems to verify the importance of gene x environment interactions in phenotypic expression. Rather than assuming that the candidate gene is not relevant because the main effect is not significant, we should be asking whether there are particular circumstances under which this gene explains a significant amount of the variance and others where it does not.

The size of the HyperGen data set provides one of the few well powered opportunities to examine interactions separately by gender. The present data suggest that using endophenotypes (here, heart rate) and evaluating interactions of genetic and environmental factors (here, anxiety) provides a valuable way to investigate and understand the complexities of pathophysiological mechanisms that may underlie components of hypertension, and may provide clues for the development of improved treatments for this complex disorder.

## Materials and Methods

### Ethics Statement

This study was conducted according to the principles expressed in the Declaration of Helsinki. The study was approved by the Institutional Review Boards of the participating studies. All patients provided written informed consent for the collection of samples and subsequent analysis. Study design and methods can be found in: Williams RR, Rao DC, Ellison RC, Arnett DK, Heiss G, Oberma A, Eckfeldt JH, Leppert MF, Province MA, Mockrin SC, Hunt SC. NHLBI Family blood Pressure Program: Methodology and Recruitment in the Hyper GEN Network. AEP 200;10:389–400. The HyperGEN Study was reviewed by the Institutional Review Boards of each site: The Institutional Review Board of the University of Alabama at Birmingham Medical School, the Institutional Review Board of Boston University School of Medicine which uses INSPIR, the Integrated Network for Subject Protection in Research, the Institutional Review Board of the University of Minnesota Medical School, the Institutional Review Board of the University of Utah Medical School, the Institutional Review Board of the University of Minnesota, and the Institutional Review Board of the University of North Carolina.”

### Cohort

The HyperGen collaboration involves four field centers of the National Heart, Lung, and Blood Institute Family Heart Study (Framingham, Minneapolis, Salt Lake City, and Forsyth County) and a fifth field center in Birmingham, Alabama. There were three groups of participants: siblings with severe and mild hypertension, an age-matched random sample of persons from the same base populations, and unmedicated adult offspring of the hypertensive siblings. The addition of this latter group permits phenotypic characterization of genetic polymorphisms associated with hypertension and intermediate phenotypes [28]. The present analyses are based on data from all three groups.

### Genome Analyses

DNA extraction and purification was done with sodium dodecylsulfate cell lysis followed by salt precipitation for protein removal, using commercial Puregene reagents (Gentra System, Inc., Minneapolis, MN). The distribution of allelic variants differed markedly by ethnicity with participants with African-American ancestry having a higher distribution of the TT genotype than participants with Caucasian ancestry. Since the number of African American subjects were relatively small (n = 208), we limited the analysis to the Caucasian participants only. Genome scans were performed by the Marshfield Mammalian Genotyping Service and the M235T AGT polymorphisms were typed in 1,960 and 2,627 women.

### Heart Rate

Heart rate was measured with a Dinamap Blood Pressure cuff model 1846 SX/P simultaneously with blood pressure. Six consecutive measurements were made, three on the left and three on the right arm. The participant rested for 5 minutes before measurement and for 30 seconds between measurements. The measure used in these analyses as the phenotype was the average HR from all six measurements.

### Anxiety and covariates

Anxiety was measured with the Spielberger trait anxiety scale [29]. This measure was chosen because it represents a stable trait rather than a transient reaction. For the purposes of these analyses, it was dichotomized into high and low anxiety groups using the upper and lower tertiles, rather than a median split, to reduce the possibility of misclassification. The covariates were age, hypertension status, smoking, alcohol consumption (ml per week), hours of television viewing (a proxy for sedentary life style), body mass index, beta blocker use (which can affect HR), and physical activity (number of blocks walked per day).

### Statistical Analyses

The number of genotyped individuals with measures of anxiety and all covariates was 987 women and 1002 men. Generalized estimating equations were used to obtain the regression coefficients and adjusted means [30], [31], specifying family as the clustering variable and adjusting for the dependence of data within families. All analyses were conducted using the software package SAS version 9.1 (SAS Institute, Cary, North Carolina). The statistical significance critera was α = 0.05.

Because of physiological differences in men and women that influence cardiac outcomes and because of differences in average heart rate in men and women in this cohort, analyses were performed separately by gender. First sex-specific associations between anxiety and heart rate and between AGTM235T genotype (MM, MT, TT) and heart rate were calculated, adjusting the regressions for beta blocker medication, age, body mass index, smoking status, alcohol consumption, number of blocks walked per day, and hours of television watched. We then regressed heart rate on AGTM235T genotype (MM, MT, TT), anxiety score and the interaction of genotype and anxiety score in gender specific models to investigate whether the association between anxiety and heart rate would be modified by AGTM235T genotype adjusting for the same covariates.
